# *Nocardia asteroides* ocular infection

**DOI:** 10.1186/1471-2334-14-S3-P2

**Published:** 2014-05-27

**Authors:** Coimbatore Subramanian Shobana, Haridas Sreekumar, Prithiviraj Aiswariya, Kanesan Panneer Selvam, Yendrembam Randhir Babu Singh, Perumal Gomathi, Palanisamy Manikandan

**Affiliations:** 1 Department of Microbiology, Dr. G.R. Damodaran College of Science, Coimbatore – 641 014, India; 2 Department of Microbiology, M R Government Arts College, Mannargudi – 614 001, India; 3 Department of Microbiology, Aravind Eye Hospital and Post Graduate Institute of Ophthalmology, Coimbatore - 641 014, India

## Background

*Nocardia asteroides* can cause ocular infection in immunocompetent and immunocompromised individuals following minor trauma. Hence present study was carried out to isolate *N. asteroides* from the patients attending a tertiary eye care hospital and to analyse the antibiotic susceptibility pattern of the *Nocardia* isolates.

## Methods

The ocular samples *viz.*, corneal swab, corneal scraping, aqueous tap and vitreous tap were processed for culture and the isolated *Nocardia* species were further confirmed for speciation by standard microbiological procedures. Antibiotic susceptibility analysis for the confirmed isolates of *N. asteroides* was performed by agar disk diffusion method following the guidelines of Clinical Laboratory Standard Institute (CLSI, 2000).

## Results

Out of 280 ocular clinical specimens collected, 25 isolates of *N. asteroides* were obtained. The rest of the pathogens included bacteria, fungi and mixed culture. The age in most of the cases yielding *Nocardia* was below 5 years (12%) and above 60 years (52%) and men (76%) were more infected than women (24%). Precisely, 17 isolates of *N. asteroides* were isolated from corneal ulcer, 5 from corneal swab and 3 from endophthalmitis. Upon analysis of antibiotic susceptibility tests the nocardial isolates were found to be more susceptible to fourth generation fluoroquinolones antibiotic family (75%), amikacin (66%), ampicillin (80%), vancomycin (50%) and norfloxacin (75%).

## Conclusion

Ocular nocardiosis remains difficult to recognize, there by leading to misdiagnosis and underestimation of its incidence.

